# Situated efficacy: FMD vaccines in France and Britain, 1930s–1960s

**DOI:** 10.1017/mdh.2025.10016

**Published:** 2025-07

**Authors:** Delphine Berdah

**Affiliations:** Université Paris-Saclay, Études sur les Sciences et les Techniques (EST), 91 400 Orsay, France

**Keywords:** Vaccines, Statistical evidence, Imaginaries and representations, Situated knowledge, Foot-and-mouth disease (FMD), Pharmaceutical industries

## Abstract

Following the trajectories of vaccines against foot-and-mouth disease (FMD) in France and Britain up to the 1960s, this paper will show how vaccine efficacy has two meanings: 1) technical – or *experimental* – which refers to test protocols and regimes of evidence, and 2) practical – or *experiential* – which refers to the experience various actors have of diseases and their direct or indirect impacts on society and the economy, as well as on representations and imaginaries they share about diseases, vaccines, and vaccination. The assessment protocols in the two countries are analysed to show how these two meanings are deeply intertwined and influence the different public policies chosen by each country. Although statistically assessed, the efficacy of the same vaccines appears situated, depending not only on regimes of evidence but also on the reality of agricultural practices, on national stock exchanges, and on various imaginaries about animal health and the absence of disease that differ between and within countries. As a consequence, this analysis reveals how public policies regarding vaccination do not always come from governmental incentives but can also emerge from private and local initiatives.

## Introduction

The history of epidemics and their control has highlighted the numerous oppositions generated by vaccines, against the social control they impose, in defiance of the public authorities or because of potential secondary side effects.^1^ The recent controversies about the efficacy of vaccines against SARS-CoV-2 illustrate well what Jeremy Ward calls *conditional critiques* ^2^ that the vaccine was developed ‘too quickly’; that there was insufficient consideration of potential side effects; that hygiene and other sanitary measures (e.g. masks, social distance, ventilation) would be more appropriate to prevent contagion. Said differently, are these vaccines actually, that is, in everyday life, safe and efficacious? What range of evidence enables assessment of their properties and their stability? It seems that all these questions require an ‘objective’ answer that should come from scientists to put an end to public fears or resistance. But since Ludwick Fleck’s work on the social construction of medical facts in the 1930s^3^ and its rediscovery in the late 1980s,^4^ numerous studies in the history and sociology of science and medicine have analysed the social dimension of illness, disease, and medical knowledge. In particular, they have shown how illnesses have both biomedical and experiential dimensions, or what qualifies as biomedical evidence is socially negotiated and interpreted.^5^ Many comparative histories of human vaccination in Western countries have been written within that frame.^6^ Perhaps the most emblematic work is Linda Bryder’s comparative history of the anti-tuberculosis vaccine BCG (Bacillus Calmette–Guérin).^7^ She shows how the data gathered by Albert Calmette, the co-founder of BCG, were bitterly criticised by British medical statisticians. According to Bryder, the opposing positions of the scientists were underpinned by ideological differences (a liberal ideology based on individual freedom of choice to be vaccinated or not) and by different approaches to disease prevention (favouring an efficient public health system with sanatoriums). Conversely, Calmette’s data were not problematic in Scandinavia, where vaccines were accepted. This ideological analysis seems to revivify Erwin Ackerknecht’s thesis, according to which liberal countries such as Britain favoured sanitary measures instead of control over bodies.^8^ However, as Peter Baldwin has pointed out, a careful comparative analysis of different diseases in various countries shows that states’ ways of controlling diseases vary widely, both within and amongst countries, between diseases and over time, according to many issues – social, economic, political, medical, or scientific.^9^ Historians of human and veterinary medicine have since confirmed his claim in fascinating accounts that analyse diverse contexts and situations.^10^ However, all of these case studies have a common ground: they all depict strong administrative structures and the state’s commitment to impose their policies, whether sanitary (mainly based on diagnosis/isolation/disinfection) or medical (based on vaccination or serum therapy). Medical policies imply a state oversight of vaccines’ safety and efficacy, no matter whether the producers of these biologicals are public institutions or private pharmaceutical companies.

Focusing on the trajectories of vaccines against foot-and-mouth disease (FMD) in France and the UK between the 1920s and the 1960s, this paper is an attempt to complete this social history of vaccinations, showing that vaccination policies are not always a top-down process emanating from the states but can also emerge as the result of private and local initiatives that eventually are endorsed by the whole country. These local initiatives are rooted in various experiences of FMD (those of farmers, veterinary practitioners, and virologists) that nourish imaginaries (i.e. the intuitions, perspectives, discourses, and utopias that accompany the design of a new technology and its uses amongst populations),^11^ which play an important role in the assessment of the vaccines’ efficacy.

FMD primarily affects cattle but also pigs. Its transmission to humans is exceptional.^12^ The disease, usually not lethal, is characterised by ulcerations on the hooves, jaws, and udders of contaminated animals that prevent them from eating, standing, and being milked. FMD thus impacts the whole agricultural economy of a country, especially since there are seven serotypes of the FMD virus, including many variants that not only provoke symptoms of diverse intensity,^13^ but also render natural immunity impossible. For all these reasons, up to the nineteenth century, the virus was rampant in Europe, and populations were used to dealing with the disease. Abigail Woods has written a complete history of FMD control in the UK. According to her, it is from the last quarter of the nineteenth century that the UK considered FMD a contagious disease, against which it was possible to establish sanitary measures. Indeed, the UK had large agricultural exploitations and was a leader in the pedigree livestock international trade. In order to maintain this lucrative trade towards countries free from FMD (such as the United States or Canada), the British government, backed by pedigree livestock farmers – often members of the House of Lords – adopted a ‘stamping out’ strategy: a sanitary policy based on the isolation and culling of contaminated animals and their contact, in exchange for compensations.^14^ Woods clearly demonstrates why the British Ministry of Agriculture, Fisheries and Food (MAFF) remained convinced that slaughter was the best and most economical solution to control the disease. She mentions serums and vaccines as continental options that the MAFF could not accept and justifies that position by ‘national differences in geography, commerce and scientific tradition’ that influenced the way researchers in the UK and the Continent judged vaccines, their risks, and their benefits.^15^ However, research for efficient vaccines against FMD in Britain engulfed massive public funds since the 1920s and led to a commercial partnership in the 1960s with the Wellcome pharmaceutical industry to produce and sell the vaccines abroad.^16^ While Abigail Woods explains this with reference to military reasons, she does not say much about how British virologists apprehended this research, settled their protocols, and assessed the vaccine’s efficacy. This untold side of the story is, however, worth analysing in comparison with another vaccinating country such as France. Indeed, in the late nineteenth century, France faced different issues. FMD was rampant, jeopardising milk production and the animal workforce.^17^ But the French government did not seem to really consider FMD a national issue: some measures of isolation and disinfection of affected premises had been enacted but without sanctions if not applied. As a consequence, many folk remedies were used to alleviate the symptoms as much as possible.^18^ Some pharmaceutical industries chose to invest in vaccine production as early as the late 1930s.^19^

The purpose of this paper is to go beyond the economic and national cultural explanations of the rejection of vaccines, to analyse the knowledge and practices developed to assess the vaccines’ efficacies in each country. There were indeed many similarities: 1) French elites – politicians, veterinarians, and scientists – shared the same conception as the MAFF about the way animal diseases should be managed; 2) both countries launched research on FMD immunity and vaccines; 3) the vaccine preparation process was similar,^20^ and French and British virologists shared the same frame of thought regarding the regime of evidence – statistical – that had to be put in place in order to assess their efficacy. However, despite these similarities, the scientists in charge of testing the vaccines did not agree on their degree of efficacy, nor on the way they had to be used as a control tool. As demonstrated by Christoph Gradmann and Jonathan Simon, sera and vaccines are biological substances, difficult to standardise, for which the preparation through the attenuation of their pathological properties needs a constant clinical evaluation to ascertain their innocuousness and efficacy.^21^ Standardising these substances requires various systems of control – scientific, industrial, and clinical – which mobilise a large range of evidence (e.g. statistical and physiological) to ascertain the homogenisation of the effects in practice. These processes have always been difficult to establish and are the result of alignment between the work done in the laboratory and the clinical observations. Moreover, in *The Invisible Industrialist*, Jean-Paul Gaudillière and Ilana Löwy analyse how standardisation possesses a double signification – a ‘restricted’ meaning that ‘denotes a formal agreement’ on the techniques, substances, or instruments to use ‘to enforce the replicability of measurements and operations’^22^ – and a ‘metaphorical meaning’, which refers to the ‘moral and social values’ that a technology posesses, which is ‘a certain understanding of the quality, the excellence or the value that gives the basis to assess and reach consensus’.^23^ This paper follows Gaudillière and Löwy to show how vaccine efficacy also possesses two meanings. The first one, technical – or *experimental* – refers to test protocols and regimes of evidence that are settled to assess the vaccines’ capacity to protect from infection. The second, practical – or *experiential* – refers to the experience various actors have of diseases and their direct or indirect consequences on economic and social exchanges, as well as on representations and imaginaries they share about vaccines and vaccination.^24^ These two meanings, experimental and experiential, historically and culturally situated, contribute to the populations’ perceptions of risks due to diseases or vaccinations. As a result, the very same vaccine could be perceived as completely efficacious by certain actors but rejected by others, both between and within nations.

This paper will analyse the issues and imaginaries underlying these scientific, political, and economic choices in France and Britain, and show how the two meanings of efficacy are deeply intertwined, explaining why France used vaccines despite their rejection by political and veterinary elites, and also why British produced vaccines that were not used inland. In its first two parts, it will focus on how the two meanings of efficacy – experimental and experiential – were characterised for each group of actors involved in the control of foot-and-mouth disease. A third part will show how, in the French case, these two meanings were aligned in favour of the absence of animal symptoms, contributing to the redefinition of the standards of efficacy, and to the generalisation of vaccines. On the contrary, in Britain, the standardisation of safety and efficacy tests discarded vaccines from their inland use, whereas they were exported to Britain’s commercial partners as control tools of the disease, redefining the conditions for trade in meat and animals on international markets.

This paper is based on the exploration of public sources, i.e. the French and British National Archives (Centre des Archives Contemporaines/CAC and Public Record Office [PRO]), private sources (from the Mérieux Institute),^25^ as well as on professional agricultural press and veterinary journals.

## In Britain: biological warfare and medical research

In Britain, research into means of immunisation against FMD emerged at the crossroads of various interests. The Treasury was looking for a less expensive solution than slaughter, and the Medical Research Council (MRC) was looking forward to developing new knowledge of viruses that could be relevant to human diseases. Thus, despite the hostility of the Ministry of Agriculture, a research station dedicated to the study of the FMD virus was established in the tuberculin production centre of Pirbright, Surrey.^26^ The study of viral properties, modes of transmission and resistance to external agents, as well as the development of typological tests, occupied most of the station’s researchers. This new knowledge was used to trace the origins of virus importations and to strengthen border controls. As it was still impossible to cultivate the virus *in vitro*, its culture *in vivo* on susceptible bovines made the implementation of large-scale or even medium-scale experiments within the station difficult. As a result, in the 1930s, only a very small number of experiments, conducted mostly with the consent of farmers on surrounding farms, sought to establish the value of serum therapy in protecting pedigree cattle.^27^ The Treasury planned to use this process to avoid expensive compensations in case of an FMD epizootic, but it was in no way a question of abandoning slaughter, the only method considered by the Ministry of Agriculture, by breeders of pedigree cattle and by veterinarians, as being able to eradicate the virus and keep the territory disease free.^28^ Indeed, as Susan D. Jones has shown, the British mapping of epidemics associated the concept of health with the absence of germs – viruses or bacteria – in a given area.^29^ Hygiene and sanitary policies were therefore the main weapons against diseases. Moreover, according to Abigail Woods, the Ministry of Agriculture and the pedigree cattle breeders shared ‘deep-seated, … not even consciously recognized’ convictions that slaughter represented a ‘virile symbol’, a ‘moralizing’ and ‘educational force’, making Britain a strong nation facing the virus enemy (often imported from abroad) while nations allowing germs to spread on their soil (like France) were seen as ‘disorganized, inefficient, ignorant and immoral’.^30^ There, the preservation of pedigree cattle through these sacrifices also referred to imaginaries related to the preservation of British aristocratic lineages and the prestige associated with them.^31^

Things changed when the hostilities of the Second World War broke out. A few months before, in 1938, a German research team led by Otto Waldmann had managed to develop an effective vaccine against FMD that could be produced on a large scale. Within the war context, the British Ministry of Defence and Her Majesty’s Cabinet feared that Nazi Germany, protected by the vaccine, would use FMD viruses as biological weapons.^32^ Brian Balmer has demonstrated how livestock diseases (such as anthrax or rinderpest) were seriously considered as effective military weapons, as they could cripple the economy of the enemy and cause food shortages.^33^ In that context, mastering the production of a vaccine against FMD seemed strategically essential. The Cabinet and the Ministry of War secretly decided to induce research in that direction, allocating massive grants to the Pirbright station. First, new buildings were set up to enable virus culture in large farm animals; then a permanent team was hired, with Ian Galloway, a veterinarian trained at the National Veterinary School of Alfort (ENVA) and at the Pasteur Institute in France, as director. The aim of the team was not only to study the value of existing vaccines but also to achieve the production of safe, reliable vaccines in Britain. Indeed, the Waldmann vaccine was a live attenuated vaccine. If the mitigation process were not carried out correctly, the vaccine could give rise to new outbreaks, especially since British bovines were particularly susceptible to FMD, as they had not been previously exposed. Similarly, the use of a vaccine with limited effectiveness could have allowed the virus to circulate in the territory, i.e. within animal bodies, and both members of the government and pedigree cattle breeders would not accept it. The experiential efficacy of a vaccine would thus have been a vaccine able to block the circulation of germs within the animal body, as imaginaries about the preservation of pedigree cattle met imaginaries about germ intruders within bodies.

In order to produce usable vaccines, the research team chose to commit to the development of standardised protocols for evaluating their innocuousness and efficacy. As Jean-Paul Gaudillière pointed out with regard to the preparation of hormonal products at *Bayer and Schering* , this standardisation was not only aimed at evaluating the effectiveness of the products, but it was also intended to monitor and control all the stages of the production process,^34^ which required a huge effort to stabilise the experimental conditions by studying all the parameters. Experiments were therefore carried out to determine which were the most optimal experimental parameters to approximate the natural conditions of infection: the length of exposure; the conditions of exposure (e.g. size of boxes, number of infected bovines, symptoms of contaminated animals); and the atmospheric and ventilation conditions of the premises.^35^ At the same time, the veterinarian William Macgregor Henderson developed an intradermal tongue test for titrating the virus on living bovines.^36^ From there, the team established a standard method for quantitatively estimating the infectiousness of viruses.^37^ The team also worked on the typification and culture of new FMD virus strains (named SAT 1, SAT 2, SAT 3, and Asia 1) and developed the concept of subtype to explain why bovines were only partially immunised against the viruses. All these results contributed to rewarding the Station with the status of the world centre of expertise on FMD viruses. On that basis, the research team sought to determine the best vaccination protocols; all the various parameters were examined in eighty experiments mobilising more than five thousand bovines.^38^ With the Cold War, the Ministry of Defence became even more concerned with achieving the production of effective vaccines in Britain. The United States and Canada shared this concern, while the Soviet Union replaced Nazi Germany as the chief threat.^39^ In a three-way alliance similar to the early Manhattan Project or to the production of antibiotics in time of war, Britain intensified its vaccine research on behalf of the three countries that did not have a laboratory offering satisfactory conditions of isolation, and invested more money into research for FMD vaccines. As Abigail Woods explains, the Department of Defence also saw this engagement as an opportunity to improve its relations with the United States.^40^ The objective was not only to protect oneself from a potential enemy attack but also to produce biological weapons in case of a hot conflict.^41^ This research had, of course, to remain confidential, and given the importance of the military stakes, it was placed under the supervision of a military officer who had to report regularly to the Ministry of Defence, whilst notifying the Ministry of Agriculture as little as possible.^42^ In addition, work to isolate the station was undertaken. Cumulatively, the work on the buildings and tensions due to military supervision led to significant delays in research, so that in the early 1950s, it was still impossible to produce vaccines on a medium scale,^43^ although many advances were made in *in vitro* culture and in local production.^44^ But as the military goal remained secret, so were the delays and difficulties.

During the winter of 1951–1952, a very serious FMD outbreak occurred. As it affected a large number of farms, strong protest movements emerged against the massive slaughter of herds. Through a press campaign, some veterinarians, small breeders, and journalists called for a more ‘modern, scientific and humane’ process: the application of vaccination, as it was carried out on the continent.^45^ However, the Ministry of Agriculture could not comply with this request for several reasons. First, Pirbright was not able to supply the vaccines, and the Ministry could not admit it because of the colossal sums invested. Second, vaccines would have had to be imported, and the Treasury was struggling to limit imports as the country was going through a financial crisis. Third, it would have been necessary to give up, at least temporarily, the pedigree cattle trade that, in addition to making the country proud, brought in many currencies, essential to the status of the country:

We must balance our external accounts. If we do not, the £ will collapse. People in this country don’t realise what this means because for centuries we have had a stable currency. (…) The collapse of the £ would mean widespread and hopeless unemployment because we should not be able to afford to buy the raw materials to keep our factories going. It would mean that people’s savings would melt away; and, finally, it would mean the disappearance of Great Britain as a power in the world.^46^

The preservation of the pedigree cattle trade seemed deeply linked with the power and prestige of the country, values that could not be negotiated. In response to the press campaign, the Ministry of Agriculture, supported by the National Farmers’ Union – on behalf of pedigree cattle breeders – insisted on the many practical limits of vaccines:^47^ 1) the duration of the immunity conferred by the vaccines was short (six months); 2) it was impossible to ensure that a vaccinated animal would not become a ‘healthy carrier’: an asymptomatic animal capable of contaminating unvaccinated cattle; and 3) the vaccines were ineffective on pigs. If the virus were to circulate in the territory, pigs would then become major propagators. The population had to trust its FMD experts; vaccines were too risky for the country.^48^ Despite this communication campaign, many small farmers kept asking for vaccines.^49^ To answer these public pressures, an expert committee, the Gowers’ Committee – named after its chairman, Sir Ernest Gowers – was set up to compare the value of the sanitary and medical policies against FMD. After hearing a large number of experts (British and continental) and representatives of agricultural and veterinary associations, the Committee concluded that the vaccines were not suitable for controlling the disease in Britain since they were not fully efficacious. This assertion was not a pretext to justify the maintenance of stamping out at all costs, as the Committee considered vaccination possible in the event of an epidemic so severe that slaughter would be impossible.^50^ It was rooted in the experience of veterinarians and large-scale farmers who adhered to this cartographic representation of epidemics and who considered virus eradication possible through a drastic control of imports, thanks to British force and will. But it was also based on the expertise developed at Pirbright, where drastic standards of safety and efficacy tests had been settled, contrary, according to Ian Galloway, to all other producing centres.^51^ Only the tests carried out at Pirbright offered, according to him, the maximum guarantee within the known experimental limits: the safety tests for a batch of vaccine were based on Henderson’s method, on eight bovines. The first six were inoculated with four dilutions of the vaccine placed at five different points on the tongue, so that twenty points could be observed for each animal, therefore amounting to 120 points in total. The dilutions were calculated to embrace the viral infecting dose for fifty per cent of the animals (i.e. the appearance of ulcerations on half of the inoculated points). If the virus had been properly attenuated, no ulcerations should be visible. To ensure that the bovines used in this safety test were susceptible to FMD, they were then tested with two other control animals with a virus of a different type than the one used to manufacture the vaccine. All should develop FMD symptoms. According to Galloway, however drastic, these tests could not guarantee that the vaccines were perfectly safe:

‘It must be remembered that while it is true that the greater the number of samples tested may be extended, a negative, in this case the absence of virus, can never be proved. Even 100 observations in our intradermal tongue test may not detect an infective level of 3% of samples positive. This may be taken as a reasonable calculated risk to take for all practical purposes for there must be a practical limit.’^52^

The conditions of clinical experimentations, which involved a limited number of animals, made the statistical value of one hundred per cent impossible to reach: as a consequence, some vaccines may therefore not have been properly attenuated but have passed safety tests. Galloway considered that continental vaccines, designed to protect animals against two or three types of FMD virus and whose safety tests did not meet Pirbright conditions, were two or three times more dangerous than monovalent vaccines. It was therefore impossible to import these vaccines without taking the risk of creating new outbreaks. Galloway was even more negative with efficacy tests. To meet British standards, three groups of eight bovines had to be inoculated with one batch of vaccine; the first group would receive the pure vaccine, the second the vaccine diluted to a quarter, the third group the vaccine diluted to a sixteenth (i.e. inoculated with 1/16^th^ of the recommended protective dose). Once the immunity was acquired, i.e. three weeks later, the bovines were tested by an injection of virus, at a dose 10,000 times higher than that contaminating fifty per cent of the animals (a fifty per cent infective dose). A week later, all animals had to be free of any lesions on the feet, even those that had received the diluted vaccine. The severity of these tests was justified by the risk of introducing a control method that could potentially worsen the epizootic. But as a consequence, it eliminated all existing vaccines, not only those produced on the continent, but also those from Pirbright!

However hard these conclusions, research continued at Pirbright, not only to maintain the knowledge and know-how related to virus culture, titration, and vaccine production, but also because British vaccines against FMD were soon intended for foreign countries, which wanted to export cattle to the UK.^53^ This remote control was perfectly in line with the British frame of thought, consisting in protecting ‘the island’ from contagion while preserving its trade by establishing quarantines and remote inspections of its borders, as it had been the case, for example, in Gibraltar or Malta in the nineteenth century, with cholera.^54^ Dangerous importations of meat or animals carrying FMD would be controlled within their own countries, thanks to British vaccines whose efficacy was guaranteed by Pirbright standards:

Anything done at Pirbright to develop vaccines and to facilitate their use abroad cannot fail to help us in the long run. Our nearness to the continent is one very good reason for our interest in the African and Asian types of virus. They could readily spread to us if introduced to the Continent because the vaccines currently in use there afford no protection against them.^55^

Used ‘abroad’, the vaccines were thus reliable tools, making it possible to keep British herds free of germs, whether attenuated (in vaccines) or not.

## In France: industrial safety and efficacy tests ‘adapted to reality’^56^

In France, the Ministry of Agriculture, like the veterinary elites such as Gaston Ramon or Emmanuel Leclainche who were involved in the management of epizootics on an international scale within the *Office International des Epizooties* (OIE) – formerly the World Organization for Animal Health – and in close contact with British experts, shared their opinion on the merits of slaughter against FMD.^57^

Emmanuel Leclainche studied veterinary medicine at the National Veterinary School of Alfort (ENVA) and was trained as a bacteriologist at the Pasteur Institute of Paris. Professor at the ENVA and then at the National Veterinary School of Toulouse, he co-founded the Toulouse Institute of Serum Therapy (IST), a pharmaceutical laboratory specialised in the production of vaccines and serums for physicians and veterinarians. He was also a member of the Advisory Committee on Epizootics (Comité Consultatif des Epizooties, CCE^58^) and the initiator and first director of the OIE. Gaston Ramon studied veterinary medicine at the ENVA and became an eminent member of the Pasteur Institute. He discovered the anatoxins (against diphtheria and tetanus), flocculation reactions, and how to boost immunity as a reaction to vaccines through the use of adjuvants. He was a member of the French Academy of Medicine and the French Academy of Science. He was also a member of the OIE and its second director. Both of them were renowned microbiologists and possessed a certain expertise on FMD: the IST sold vaccines against the disease, and Ramon had investigated FMD vaccination at the Pasteur Institute (with no much support from the Direction nor much success)^59^. But despite their experience with vaccines, both veterinarians were convinced that slaughter was the best way to eliminate the disease, and they made numerous communications at the CCE, in the Senate, or at the Assembly in that direction. Despite the authority of these two veterinarians, the French government refused to implement the sanitary policy not only because of the amount of compensation required, but also because most farm work relied on bovines (mechanisation was still limited and horses were expensive and scarce^60^). A mass culling of farm animals would have jeopardised French agriculture in general. In addition, slaughter had no support from veterinarians. According to their professional union, veterinarians were too attached to their liberal status to mimic British veterinarians and get involved with a sanitary policy that would provide them with a status close to that of civil servants.^61^ As a result, the isolation and disinfection policy was poorly implemented, so that FMD viruses circulated freely in the country. Animal owners were used to having contaminated animals recovering from FMD, and they generally considered this disease a mild condition (apart from a few exceptional episodes). They treated their sick animals with various curative procedures inherited from their families or purchased from healers, peddlers or pharmacies. These products were most often made from antiseptics such as vinegar, thyme, or wild thyme, to avoid the most problematic complications (e.g. myocarditis, metritis, abortions, mastitis, enteritis, neonatal mortality). Farmers could also speed up the infection process to get rid of the disease more quickly, by moistening the gums of their animals with contaminated saliva, a practice called aphtisation. Finally, the sanitary veterinarians (working for French departments) and certain veterinary pharmaceutical manufacturers had developed a process of passive immunisation, called serum therapy, which consisted of inoculating the animals with the serum of convalescent animals. Despite its short-term protection, this technique was very popular amongst farmers and liberal veterinarians in the 1930s.^62^ The immunisation processes – whether active or passive – referred to the bacteriological culture characteristic of a form of French expertise, supported by the Pasteur Institute and which was shared by veterinarians, either due to their training^63^ or to their association with Pastorians in various public and private enterprises.^64^ But it also fitted very well with a pre-bacteriological *style of thought* ^65^ still present in France, a sort of *holistic* representation of illness that integrated germs (viruses and bacteria) together with humans and animals within their environment.^66^ Getting rid of infections could only be done, therefore, according to the Pastorians, by replacing pathogenic germs with harmless germs (killed or attenuated) within individuals, through vaccines.^67^ This replacement ideology has to be understood in the same manner that the French nineteenth-century botanists Augustin and Alphonse De Candolle spoke of ‘nature’s war’ or the ‘plant war’ to describe plants’ competition to occupy the same environment. In the case of France, this ecological representation of the germs’ relations within an organism met the possibility to experiment with alternative means of control of animal diseases, and the farmers’ aspirations to preserve animal bodies as much as possible. Various bacteriological laboratories which marketed serums and vaccines, such as the Pasteur Institutes in Lille and Paris, the Roger Bellon Laboratories, the Laboratory of serum therapy of L’Aigle, or the Bacteriological Institute of Tours, to name but a few, therefore encountered many successes with farmers. Amongst these laboratories, the Mérieux Institute (IM) in Lyon, sold so much serum against FMD that it decided after the war to join forces with the Toulouse Institute of Serum Therapy (IST) to produce on a large scale a vaccine against FMD. The process developed by Otto Waldmann supposed an *in vivo* culture of the virus in bovines. Once the characteristic ulcers reached a peak, the animals were killed, their lingual epithelium removed and ground, and then the virus was mitigated. The IM and the IST, therefore, founded the French Institute for Foot-and-Mouth Disease (IFFA) in Lyon, a vaccine production centre large enough to serve as a slaughterhouse. The IFFA sold trivalent vaccines (to the State, the IM, and the IST), made by the successive preparation of monovalent vaccines – each active against one type of virus – that were combined at the time of injection. The culture of each viral strain had to be done one after the other to avoid any contamination.^68^ The yields were very low, as the slaughter of a 500-kg bovine led to the production of thirty to forty grams of virus, enough to produce fifty doses of vaccine.^69^ This forced the IFFA to slaughter an ever-increasing number of animals to achieve industrial production, which was not without problems, in particular, concerning the sale of meat.^70^ Once produced, vaccine batches underwent three safety tests:^71^ a physico-chemical test (the pH of the vaccine had to be close to nine); a bacteriological test to detect potential bacterial impurities; and a biological test on three bovines. After being inoculated with the vaccine, these animals were placed under observation for fifteen days, in isolation. A herdsman – not a veterinarian – was responsible for observing any visible symptoms that would lead to the rejection of the batch of vaccine. Two weeks after these tests, the efficacy test was performed on the three animals plus a fourth, as a control (all of them were infected with a virulent FMD virus). The control animal had to show symptoms of generalised FMD. Without the possibility of titrating the virus or the vaccine, the only method of analysing the efficacy of the vaccine was therefore the clinical comparison of the biological modifications caused by the inoculation of the virus in the animals. To this end, a veterinarian performed a clinical examination: if no reaction was observed or, failing that, if only a local ulcer appeared, the vaccine was considered ‘good’. In all other cases – multiple mouth ulcers, ‘thermal reaction’, generalised foot lesions – the vaccine was rejected.^72^ The results of these tests mobilised a register of clinical evidence that depended considerably on the susceptibility of bovines to the FMD virus. According to Maurice Fedida and his collaborators, employees at the Laboratory of Animal Virology – where vaccines manufactured by the IFFA were controlled in the 1970s – only fifty per cent of the vaccines designed in the 1950s were considered ‘good’ at the end of these efficacy tests.^73^ Even if this testimony probably aimed to reinforce, by contrast, the quality of current vaccines, it nevertheless allows a glimpse of what the conditions of production and evaluation of vaccines were like in the early 1950s.

Between 1951 and 1953, a very strong epizootic broke out, and isolation and the suspension of fairs and markets, as well as vaccination around outbreaks (‘ring vaccination’ operations), were insufficient to control contagion.^74^ As a result, individual vaccine demands skyrocketed as farmers attempted to save animals that had not yet been affected. Vaccines were sent to veterinarians in an emergency, often too late.^75^ Worse, new variants appeared, rendering obsolete the vaccines that had first succeeded in protecting some animals.^76^ All agricultural work seemed paralysed, and food shortages increased. Faced with the urgency of the situation, the Minister of Agriculture opposed the recommendations of the French Academy of Science^77^ and restored the circulation of bovines on the territory to avoid the financial crisis caused by the cessation of all commercial activities.^78^ The Ministry of Agriculture mobilised the army to help bring in the crops, as too many animals were unable to work,^79^ and ordered them to carry out trials of any possible curative treatments. This order reveals a significant cleavage: on the one hand, liberal veterinarians and breeders, depending on animal traction that looked for individual protection of bovines through any means of preservation. On the other hand, the veterinary scientific elites members of prestigious academies demanded the culling of herds as the only possible means of control.^80^

From the farmers’ view, slaughter was even more unpopular than isolation and the suspension of fairs and markets, because of their experience of the disease as a mild disease. A large majority of them used bovines to work the fields. The recovery of their animals was important as bovines were not easily replaced. Training them to work under the yoke took time and presupposed that the farmer already had an animal accustomed to working in this way, which ‘guided’ the young cattle in learning farm work. The slaughter of two animals was often simply impossible for these farms. For dairy farmers, this was more or less the same, as compensation would not cover the loss of their animals. Faced with such a reality where the culling of animals could jeopardise many farms, farmers were in favour of vaccines because they deemed any animal spared or suffering from mild symptoms of the disease a prophylactic success.^81^ Liberal veterinarians shared the same point of view, as their motto, ‘first, do no harm’, involved caring for animals – or at least sparing them as much as possible.^82^ Their representatives in Parliament asked the government to promote vaccine production to meet national needs.^83^ But for the veterinary elites, as for the Ministry of Agriculture, which doubted the real capacity of vaccines to control an epizootic, it was unthinkable to endorse this medical policy as long as vaccines lacked a real assessment of their efficacy, carried out by an independent laboratory.^84^ Unfortunately, the only public veterinary laboratory (at the ENVA) lacked the means to fulfil this role, and the Ministry of Agriculture did not plan to modify this situation, showing that FMD vaccines were not a national priority.

## Aligning experimental and experiential efficacies, generalising vaccination

The IM and the IST sought to respond to these objections while forging new commercial partnerships with liberal veterinarians. They reinvested the profits from the sales of vaccines from the 1952 epizootic in new *in vitro* culture systems^85^ and funded the creation of an ‘independent’ laboratory to control all the foot-and-mouth disease vaccines produced in France, including the IFFA’s. This laboratory – named the Veterinary Research Laboratory (VRL)^86^ – operated thanks to the IFFA’s payment of two per cent of its annual profits.^87^ In the 1950s, the international reputation of Pirbright researchers was very important, and their standards legitimising the rejection of vaccines in the UK were, of course, known to the director of the VRL, the veterinarian Félix Lucam, and to his team. However, these standards were incompatible with the industrial production developed by the IFFA and the other laboratories manufacturing FMD vaccines because their safety and efficacy tests required the use of too many animals. The VRL then adopted other standards, ‘rapid, easy to learn and cost-effective’, thus compatible with industrial production.^88^ Nevertheless, to legitimate this choice, the French researchers referred to the statistical tables published by Henderson, which showed how even the most drastic safety controls left a chance of missing a badly attenuated batch. But if this assertion based on statistical evidence justified the vaccine’s rejection in Britain, in France, on the contrary, it made acceptable the existence of a margin of uncertainty for safety and efficacy tests. Two types of tests were therefore performed. The more rigorous of the two involved a larger number of animals but was applied less often, *‘*once in* *10 or 15* *vaccine preparations**’:^89^ each valence was tested on eight bovines (plus four as controls that should develop generalised FMD). The vaccinated animals were clinically examined and the results converted into statistical values, in an operation masking the clinical variabilities.^90^ The vaccines were then classified according to the lesions observed, from ‘authorised’ – if no lesion was observed – or ‘acceptable’ – if only one animal was clinically affected – to ‘rejected’ – if two or more animals were affected. ‘In the worst case’, Félix Lucam and his team estimated that the vaccines were at least eighty per cent effective, which they considered ‘quite good’, consequently accepting in practice a twenty per cent failure rate.^91^ Despite its approximate nature, this first type of control was still considered too costly in terms of bovines to control the industrial production of IFFA. Most of the time, a second type of control was performed on two to four bovines (plus one or two as controls). If all of them, or three out of four, or two out of three animals resisted the virulent test, the vaccine was accepted. If one out of two, or two out of three or four bovines did not resist, the batch of vaccine was not destroyed, but new tests were performed. It was only in this second case, when ‘the same results or worse’ were obtained, that the vaccine was rejected.^92^ Otherwise, the vaccine was considered sufficiently efficacious and marketed. Of course, the researchers at the VRL knew that these tests involved too few animals to be statistically significant. Nevertheless, they justified their practices by adding up all the tests carried out as if they had tested only one batch, in defiance of all statistical rules:

‘Results interpretation. Safety control: out of 271 batches of vaccine checked, none proved to be virulent. We noted above the fairly large degree of uncertainty that surrounds each control. But now consider all of those 271 vaccine lots. Since these are similar materials, we can very well imagine them gathered in a single batch. For this single batch, we used 542 bovines which each received 20 samples. We have therefore, in total, tested 542 x 20 = 10,840 samples from this single batch. Since none of these samples proved to be virulent, we can apply the formula (1 – p)n = P, which made it possible to establish Table I and in which n is the number of samples tested and which all were shown to be avirulent, p is the maximum possible percentage of those which, nevertheless, in the entire batch of vaccine could be virulent, and P the probability of missing the latter.^93^ We find, under these conditions, that in our supposed batch, there could only be at most 0.003% virulent samples with a one in twenty chance of not having been able to detect them. […] It follows that if, at the time of each test, the interpretation of the result must be subject to reserve, the accumulation of tests makes it possible, *a posteriori*, to get a fairly exact idea of the average value of an important production.’^94^

According to these researchers, this series of approximations was justified by the industrial nature of the production and the controls to be carried out. Nevertheless, it helped to disseminate a numerical value that made the uncertainties surrounding the controls invisible, leading them to demonstrate that the vaccines produced in France were completely (99.997%) safe, and their efficacy foolproof. It was not a question of a lack of knowledge of the statistics (as the reference to Henderson’s statistical analysis is explicit). The logic underlying the implementation of these tests has to do with what Nathalie Jas describes as a principle of ‘adaptation to reality’ when she analyses the implementation of the acceptable daily intake tool by the WHO/FAO Committee on Food Additives in the 1950s–1960s.^95^ She shows how, knowing well that there are no threshold doses below which carcinogens would not endanger public health, the members of this committee legitimised the addition of carcinogens to food because their use was considered essential for the community as a whole. Similarly, the VRL knew perfectly well that the standards used in France were not statistically capable of guaranteeing one hundred per cent safety and efficacy. But their results approached the experiential efficacy of many farmers and veterinarians, who needed to see their animals survive FMD and avoid the slaughter requested by the French government. In this social and cultural context, a one hundred per cent effective vaccine was a vaccine capable of justifying the non-application of culling measures, even if this meant that some animals were poorly protected.

However, this was not true in all regions of France. In Finistère, the breeders who traded with Britain had decided to slaughter as soon as the first outbreaks appeared, to isolate contaminated farms and to control all the movements of the population within the department.^96^ This experience of eradication relied entirely on farmers, with the help of the local authorities, who compensated for the losses endured. Everyone agreed that this experience had been a frank success. For the Ministry of Agriculture, it was the confirmation that the sanitary policy was the solution. However, it was impossible to make it mandatory as the government was financially involved in very costly operations of the eradication of bovine tuberculosis.^97^ To protect the FMD vaccines market, the Director of the Mérieux Institute, Charles Mérieux, contacted liberal veterinarians. They indeed had no interest in seeing the sanitary policy against FMD generalised to the whole country. First, their involvement was not required either for the diagnostic or the application of the sanitary measures (as it was shown by the Finistère experiment). Second, they would lose a great number of their clients, as the vaccines, sera, or treatments against the disease would become useless or forbidden. Charles Mérieux was a close relative of Marcel Quentin, the President of the National Veterinary Union. He knew that liberal practitioners were discussing with farmers, breeders’ associations, and unions (such as the Confédération Nationale de l’Elevage, the Fédération Nationale des Groupements de Défense Sanitaire, or the Union Nationale des Coopératives Laitières) about the possibility of setting up vaccinations on a large scale.^98^ Charles Mérieux developed an alliance with the National Veterinary Union to establish partnerships with breeders opposed to slaughter. This led to vaccination contracts linking the industries producing vaccines to veterinarians and farmers for a period of five years. The settlement of such a partnership relied entirely on the relations liberal veterinarians had with their clients or with agricultural cooperatives. The cost of the prophylaxis would rest on farmers, but with negotiated prices for vaccines and veterinary inoculation, it would be cheaper than the consequences of eradication. Besides, the liberal sensibility of veterinarians (their ability to set the price of their services) was spared: their honoraria varied according to their local conditions of exercise (the terrain, the animal dispersion, and the number of animals on the farm).^99^ Vaccination operations and their annual boosters guaranteed the presence of veterinarians in the herds, the ‘syringe’ appearing as a powerful form of technical mediation^100^ between vets and farmers. These ‘vaccination contracts’ linking industrialists, farmers, and liberal veterinarians were not just a simple commercial operation for industrialists. They also contributed to aligning experimental efficacy with experiential efficacy, with the continuous improvement of vaccines. At the end, the government found itself with the setup of a medical policy with no civil servants or public money involved. Farmers interested in these measures saved themselves the losses caused by the disease, and veterinarians gained a regular income and the loyalty of their customers. Manufacturers, supported by regular purchases of vaccines and having access to large-scale safety and efficacy tests carried out through the use of vaccines in practice, increased and improved their production capacities. Eventually, the administrative arrangements were aligned with the experience of farmers who had freely chosen to subscribe to vaccination contracts: in 1961, vaccination became mandatory in France, slaughter being kept as a secondary tool, in case of an outbreak of FMD.^101^

## Conclusion

As Joan Fujimura pointed out, ‘technology alone cannot make problems doable’. She argues that ‘doability is conceptualised as the alignment of several levels of work organization’ – experiments, the laboratory, and the social world.^102^ The articulation of tasks between these three levels of work organisation makes problems doable. Following Fujimura, this paper has shown the tinkering of the VRL scientists in order to align these three levels and produce vaccines that are one hundred per cent efficient. Indeed, although falling under the same methods of preparation of FMD vaccines, and therefore possessing an experimental efficacy that can be considered similar in France and Britain, the experts on their efficacy in the two countries reached opposite conclusions. In Britain, techniques for the standardisation of preparation, inoculation, and evaluation of vaccines, such as the register of statistical evidence mobilised, made it possible to reject all vaccines, including those produced in the country, because none of them could statistically achieve one hundred per cent efficacy. The degree of uncertainty, even small statistically, reduced the possibility of using the vaccines in practice, as it objectivised the risk of immune escape and made possible an estimation of the economic losses and of the losses in power and prestige that Britain would endure. But it was also impossible for vaccines to meet the experiential efficacy required by British members of the Government and pedigree cattle breeders, with no germs inside the animal body. Indeed, vaccinated cattle appeared as healthy carriers, potentially dangerous for unvaccinated animals. Nathalie Jas showed how the construction of risk regulation tools – here, the statistical evaluation of the efficacy of vaccines according to the Henderson protocol – goes through a series of accommodations which ultimately make public health a relative issue, dependent on the need to adapt to reality.^103^ The British reality referred not only to an economic-political logic, aimed at preserving very lucrative export markets that benefitted the reputation of the country, but also to the imaginaries associating hygiene, body control, culture, and power of a nation, so that to be *effective*, a vaccine would have had to *eradicate* the germ responsible for the disease, which the FMD vaccines could not do. As a consequence of that experiential effectiveness, the experimental effectiveness was statistically impossible to reach, legitimating the practical reluctance to use vaccines. On the other hand, in France, the experimental efficacy of vaccines was articulated with the experiential efficacy of breeders who were looking for a way not to suffer from the disease’s effects. Perfectly adapted to the reality of breeders in the 1950s and 1960s, French vaccines imposed themselves even though they only imperfectly protected the entire national herd. As Loconto and Demortain argued, standards do not necessarily come from the top. They also emerge from diversity, through the appropriation of the possible applications of these standards, so that uses determine effectiveness.^104^ French vaccines, therefore, being more and more used, have gradually improved their efficacy, both through the improvement of technical processes and through the coverage effect of a greater number of vaccinated animals. This double improvement contributed to the success of vaccination campaigns up to the modification of animal disease control standards in France, through the establishment of a compulsory medical policy against FMD in 1961. Thus, the example of FMD vaccination in France clearly demonstrates that public policies can also emerge from local and private initiatives, without any intervention by the state.

## Footnotes

1 See, for example: Stuart Blume , ‘Anti-vaccination Movements and their Interpretations’, Social Science and Medicine, 62, 3 (2006), 628–642 16039769 10.1016/j.socscimed.2005.06.020; Stuart Blume , Immunization: How Vaccines Became Controversial (London: Reaktion Books, 2017); Christian Bonah and Philippe Menut , 2004, ‘BCG Vaccination Around 1930: Dangerous Experiment or Established Prevention? Debates in France and Germany’, in Volker Roelcke and Giovanni Maio (eds.), Twentieth Century Ethics of Human Subjects Research: Historical Perspectives on Values, Practices, and Regulations *(* Stuttgart: Franz Steiner Verlag, 2004), 111–127 ; Deborah Brunton , The Politics of Vaccination: Practice and Policy in England, Wales, Ireland, and Scotland, 1800-1874 (Woodbridge: Boydell & Brewer, University of Rochester Press, 2008); Nadja Durbach , Bodily Matters: the Anti-Vaccination Movement in England, 1853–1907 (Durham and London: Duke University Press, 2005); Jean-Baptiste Fressoz , ‘Le vaccin et ses simulacres: instaurer un être pour gérer une population, 1800-1865’, Tracés, 21 (2001), 77–108 ; Jean-Baptiste Fressoz, L’apocalypse joyeuse. Une histoire du risque technologique (Paris: Seuil, 2012); Christoph Gradmann , ‘Locating Therapeutic Vaccines in 19^th^ Century History’, Science in Context, 21, 2 (2008), 145–160 18831134 10.1017/s026988970800166x; Ilana Löwy , ‘Les politiques de vaccination au Brésil: entre science, santé publique et contrôle social’, Sciences Sociales et Santé, 27, 3 (2009), 105–134; Dorothy Porter and Roy Porter , ‘The Politics of Prevention: Anti-Vaccinationism and Public Health in Nineteenth-Century England’, Medical History, 32, 3 (1988), 231–252 3063903 10.1017/s0025727300048225PMC1139881; Didier Torny , ‘Politiques vaccinales’, in F. Bourdillon , Traité de prévention (Paris: Flammarion, 2009), 206–211 .

2

Jeremy K. Ward , ‘La critique vaccinale au temps du vaccin contre la grippe A (H1N1). Comparer les comparaisons’, Sciences Sociales et Santé, 4, 35 (2017), 37–59 .

3 Ludwick Fleck, *Genèse et développement d’un fait scientifique* (translated into French by Nathalie Jas) (Paris: Les belles lettres, 2005 [1935]).

4

Ilana Löwy , ‘Quantification in Science and Cognition Circa 1937. A Newly Discovered Text of Ludwik Fleck’, Science in Context, 2, 2 (1988), 345–355 11622315 10.1017/s0269889700000648.

5 For a review, see: Peter Conrad and Kristin K. Barker , ‘The Social Construction of Illness: Key Insights and Policy Implications’, Journal of Health and Social Behavior, 51, 1 (2010), S67–S79 20943584 10.1177/0022146510383495.

6 See, for example: Anne Hagen Berg and Stuart S. Blume , ‘Reasonable Grounds? The Delayed Introduction of MMR Vaccine in Denmark and the Netherlands, 1977-1987’, Medical History, 64, 3 (2020), 355–373 .

7

Linda Bryder , ‘“We Shall Not Find Salvation in Inoculation”: BCG Vaccination in Scandinavia, Britain and the USA, 1921-1960’, Social Science & Medicine, 49 (1999), 1157–1167 10501638 10.1016/s0277-9536(99)00157-4.

8

Erwin Ackerknecht , ‘Anticontagionism between 1821 and 1867’, Bulletin of the History of Medicine, 22 (1948), 562–593 18106184 .

9

Peter Baldwin , Contagion and the State in Europe, 1830-1930 (Los Angeles: University of California, 1999).

10 To name but a few: Christine Holmberg , Stuart Blume and Paul Greenough , The Politics of Vaccination. A Global History (Manchester: Manchester University Press, 2017); Volker Hess , ‘The Administrative Stabilization of Vaccines: Regulating the Diphtheria Antitoxin in France and Germany, 1894-1900’, Science in Context, 21, 2 (2008), 201–227 18831137 10.1017/s0269889708001695; E.P. Hennock , ‘Vaccination Policy Against Smallpox, 1835-1914: A Comparison of England with Prussia and Imperial Germany’, Social History of Medicine, 11, 1 (1998), 49–71 11620154 10.1093/shm/11.1.49; Mikio Kimura and Harumi Kuno-Sakai , ‘Pertussis Vaccines in Japan – A Clue Toward Understanding of Japanese Attitudes to Vaccines, Journal of Tropical Pediatrics, 37, 1 (1991), 45–47 2023303 10.1093/tropej/37.1.45.

11

Patrice Flichy , ‘La place de l’imaginaire dans l’action technique. Le cas de l’Internet.’, Réseaux, 2001/5, n°109, 52–73 .

12 For example, when veterinarians hurt themselves with medical instruments contaminated by the animal virus (transmission would require contamination by a blood secretion).

13 They range from mild symptoms to death, depending on both the nature of the variants and the individual reactions of the contaminated animals.

14

Abigail Woods , A Manufactured Plague? The History of Foot-and-Mouth Disease in Britain (London, Sterling VA: Earthscan, 2004).

15 Woods, *op. cit.* (note 14), 99.

16 Public Record Office (PRO)/MAF 255/891: Animal Health: FMD, 1955 – 1961: op de G. Bishop du 22/12/1960: ‘The collaboration between FMD Institute, Pirbright and the Wellcome Foundation to produce FMD vaccines for use abroad.’

17

Jean-Luc Mayaud , La petite exploitation rurale triomphante, France XIX^e^ siècle (Paris: Belin, 2000); Ronald Hubscher , ‘La petite exploitation en France: reproduction et compétitivité, fin XIX^e^ – début XX^e^’, Annales, 40, 1 (1985), 3–34 .

18

Delphine Berdah , ‘Serum Therapy Against FMD and the Development of the French Veterinary Profession in the 1930s’, Review of Agricultural, Food and Environmental Studies, 102, 2 (2021), 151–169 .

19

M. Lombard , P.P. Pastoret & A.M. Moulin , ‘A Brief History of Vaccines and Vaccination’, Revue Scientifique et Technique de l’OIE, 26, 1 (2007), 29–48 10.20506/rst.26.1.172417633292.

20 The *in vivo* and *in vitro* culture methods may have differed at some times, but the viruses were attenuated using the same process.

21

Christoph Gradmann and Jonathan Simon (eds.), Evaluating and Standardizing Therapeutic Agents 1890–1950 (Basingstoke: Palgrave Macmillan, 2010).

22

Jean-Paul Gaudillière and Ilana Löwy , *The Invisible Industrialist. Manufacture and the Construction of Scientific Knowledge* (Houndmills, Basingstoke, Hampshier: Palgrave MacMillan, 1998), 10 .

23

Jean-Paul Gaudillière , ‘The Visible Industrialist: Standards and the Manufacture of Sex Hormones’, in Christoph Gradmann and Jonathan Simon (eds), Evaluating and Standardizing Therapeutic Agents, 1890–1950. Science, Technology and Medicine in Modern History (Houndmills, Basingstoke, Hampshier: Palgrave MacMillan, 2010), 174–201, 175.

24 This experiential meaning of efficacy differs from ‘effectiveness’: a term used to characterise the gap between the efficacy of a drug assessed on randomised control trials and its effects in real conditions. Thus, effectiveness depends on the behaviour of both patients and physicians regarding the drug, on the observational studies that give different data than a random study, on the health care characteristics and on individual factors (e.g. biological, genetic, nutritional, environmental). See: Clementine Nordon *et al.*, ‘The “Efficacy-Effectiveness Gap”: Historical Background and Current Conceptualization’, Value Health, 19, 1(2016), 75–81 26797239 10.1016/j.jval.2015.09.2938.

25 The archives of the French Institute on FMD (*Institut Français de la Fièvre Aphteuse*, or IFFA) are stored at Marcel Mérieux’s Foundation.

26 Tuberculin is a tuberculosis diagnostic substance.

27 In contrast to a vaccine prepared from a bacterium or a virus killed or attenuated, a serum is the liquid that separates when the blood coagulates. Collected from convalescent animals, the serum contains antibodies against the microbe responsible for a disease, and can therefore protect a healthy animal that has not been in contact with the virus. The drawback to sera is that the conferred immunity is very short-lived and therefore useless on a long-term basis.

28 PRO/T 161/886: Merrick R. Burrell *et al., Report of the Sub-Committee Appointed to Review the Work of Foot-and-Mouth Disease Research Committee, as adopted by the animal diseases and animal nutrition committees on the 11 July and by the Council itself on the 19 July 1933, 1933*, 5.

29

Susan D. Jones , ‘Mapping a Zoonotic Disease. Anglo-American Efforts to Control Bovine Tuberculosis Before World War I’, Osiris, 19 (2004), 133–148 15478271 10.1086/649398.

30 Woods, *op. cit.* (note 14), 100–101.

31

Delphine Berdah and Camille Noûs , ‘Veterinary Expertise, Public Health and Animal Contagion. The Control of Bovine Tuberculosis in France and Britain, 1860-1960’ in Axel Huentelmann , Christian Jaser , Mieke Roscher and Nadir Weber (eds.), Animals and Epidemics in Historical Perspective (Berlin: Böhlau-Verlag, 2023).

32 PRO/MAF 189/326: MAF, Foot-and-Mouth Disease Research Committee, *The Following Translation of a Statement Made by Müssemier, Representing Germany, at the Recent Meeting of the Office International des Epizooties, is Circulated for the Information of the Members*, 2^nd^ June 1938, 3.

33

Brian Balmer , Britain and Biological Warfare. Expert Advice and Science Policy, 1930-1965 (New York, Palgrave Macmillan, 2001).

34 Gaudillière, *op. cit.* (note 23), 181–182.

35 PRO/MAF 33/35: *Progress Report, Pirbright, 1941-1942*, 1.

36 Titration is a method for analysing the titre of a substance (i.e. the ratio of the mass of a dissolved body to the mass of the solution). See: William M. Henderson, ‘The Quantitative Study of Foot-and-Mouth Disease Virus’, *Agricultural Research Council Report*, His Majesty’s Stationery Office, S. n^o^ 8, 1949, quoted by Edward Boden , ‘Sir William Macgregor Henderson’, Biographical Memoirs of Fellows of the Royal Society, 50 (2004), 133–146, 13815754472 10.1098/rsbm.2004.0010.

37

William M. Henderson , ‘Significance of Tests for Non-Infectivity of Foot-and-Mouth Disease Vaccines’, Journal of Hygiene, 50, 2 (1952), 195–208 14938533 10.1017/s0022172400019549PMC2235112.

38 PRO/MAF 117/394: Ian Galloway, *Draft Report for Years*, t. 1, 1935-1939; *id., Draft Report for Years*, t. 2, 1939-1952, Research Institute, Pirbright, 16.

39 PRO/WO 291/1421: R.F. Crampton, Secret mémorandum, Army Operational Research Group, *The Susceptibility of UK Agriculture to Attack by the Viruses of Foot-and-Mouth Disease*, 1954.

40 Woods, *op. cit.* (note 14), 88.

41 PRO/MAF 250/163: Minutes from Sir Thomas Dalling, by C.H.M Wilcox, 12 December 1951.

42 PRO/MAF 250/163: Confidential letter from William K. Slater to Crawford, 8^th^ August 1952.

43 PRO/MAF 117/394: Ian Galloway, *Comments on Some of the Technical and Other Aspects of Observations and Conclusions in the Report of the Departmental Committee on Foot-And-Mouth Disease on the Institute’s Extension Programme in Relation to Vaccine Production*, 5^th^ October 1954, 1.

44 Adaptation of the *in vitro* culture of Hermann Frenkel, on bovine tongue epithelium, as well as on avian, murine, porcine and bovine embryonic tissues. See: PRO/MAF 35/871: *Report for 1953-1955*, Research Institute, Pirbright, Surrey, 5.

45 Woods, *op. cit.* (note 14), 95.

46 PRO/MAF 255/112: ‘General Economic Situation. Notes for Sir Thomas Dugdale’, 16^th^ May 1952, 2.

47 PRO/MAF 33/944: *Draft for Minister’s Signature. To Be Addressed to the Treasury. Foot-and-Mouth Disease*, n.d., 6.

48 PRO/MAF 255/174: Sir James Turner, ‘The Battle Against Foot-and-Mouth Disease. Why the Slaughter of the Innocents Must Go On’, *News of the World*, 11^th^ May 1952.

49 Woods, *op. cit.* (note 14), 93, 100–101.

50 PRO/MAF 35/872: *Committee on FMD (Gowers Committee). General Recommendations, 1954-1955.*

51 PRO/MAF 255/174: Ian A. Galloway, ‘Foot-and-Mouth Disease’, *British Agricultural Bulletin*, 5, 20 (1952), 67–74, 69 and 73; PRO/MAF 117/394: Ian Galloway, *Draft Report for Years*, t. 1, 1935-1939; *id., Draft Report for Years*, t. 2, 1939-1952, Research Institute, Pirbright, 18.

52 PRO/MAF 117/394: Research Institute, Pirbright. Ian Galloway, *Draft Report for Years*, t.1, 1935-1939; *Draft Report for Years*, t. 2, 1939-1952, Research Institute, Pirbright, t.2, 17.

53 PRO/MAF 35/872: *Committee on FMD (Gowers Committee). General Recommendations, 1954-1955*, House of Lords, 20^th^ April 1955.

54

Patrice Bourdelais , Epidemics Laid Low. A History of What Happened in Rich Countries (Baltimore: Johns Hopkins University Press, 2006), 65 .

55 PRO/MAF 255/891: Animal Health: FMD, 1955-1961: Letter from Q. Hailsham to Christopher Soames, 24^th^ November 1960.

56 The terms come from Nathalie Jas , ‘Adapting to ‘Reality’: The Emergence of an International Expertise on Food Additives and Contaminants in the 1950s and Early 1960s’, in Soraya Boudia and Nathalie Jas (eds.), Toxicants, Health and Regulation since 1945 (London: Routledge, 2013), 47–70 .

57
*Journal Officiel* (JO), *Débats parlementaires, Chambre des Députés*, 4 juin 1937, 1789-1793: Discussion, après demande de discussion immédiate, d’un projet de loi relatif à la prophylaxie de la fièvre aphteuse, séance du 3 juin 1937. See also: JO, *Documents parlementaires, Chambre des Députés*, 3 juin 1937, 742–743: Annexe n°2521: Rapport fait au nom de la commission d’agriculture chargée d’examiner le projet de loi relatif à la prophylaxie de la fièvre aphteuse, par M. Albert Daille, Député.

58 The CCE’s task was to advise the Ministry of Agriculture on the policy to follow to control contagious animal diseases.

59 Private archives of the Pasteur Institute (AIP): Fonds Gaston Ramon (GR)/Boîte 20.

60

GREA (Groupe de recherche ethnologique de l’Anjou), Paroles et parcours de paysans. Nous avons cru au progrès (Paris: L’Harmattan, 1996).

61

Conseil Général Vétérinaire, Histoire des Services Vétérinaires Français (Paris: Association Amicale du Conseil Général Vétérinaire, 2006), 50 .

62 Berdah, *op. cit.* (note 18).

63

Delphine Berdah , ‘Entre scientifisation et travail de frontières: les transformations des savoirs vétérinaires en France, XIXe – XIXe siècles.’, Revue d’Histoire Moderne et Contemporaine, 59, 4 (2012), 51–96 .

64

Maurice Cassier , ‘Appropriation and Commercialization of the Pasteur Anthrax Vaccine’, Studies in History and Philosophy of Science Part C. Studies in History and Philosophy of Biological and Biomedical Sciences, 36, 4 (2005), 722–742 10.1016/j.shpsc.2005.09.00416337558; Berdah, *op. cit* (note 18).

65 Fleck, *op. cit.* (note 3).

66

Anne-Marie Moulin , ‘The Defended Body’ in Roger Cooter and John Pickstone (eds), Companion to Medicine in the Twentieth Century (London: Routledge, 2002), 385–398 ; John Andrew Mendelsohn , ‘Like All That Lives’: Biology, Medicine and Bacteria in the Age of Pasteur and Koch’, History and Philosophy of the Life Sciences, 24, 1 (2002), 3–36 12664951 10.1080/03919710210001714293.

67

Voir Gerald Geison , The Private Science of Louis Pasteur (Princeton, NJ: Princeton University Press, 1995), 163 10.1111/j.1600-0498.1998.tb00418.x11619802; Berdah, *op. cit.* (note 31).

68 Centre des Archives Contemporaines (CAC): 19880158/80: Section permanente du Comité consultatif des épizooties, séance du 7 juillet 1949.

69 Marcel Mérieux Foundation (FMM)/Personal archives of Dr Mérieux (APDM): Manuscript about the history of the Mérieux Institute, no author, n.d., 58.

70 FMM/APDM: Charles Mérieux, *Une certaine idée de la biologie*, autobiography, n.d., 30.

71 Maurice Viel, ‘L’Institut français de la fièvre aphteuse et la préparation du vaccin antiaphteux de Vallée-Schmidt-Waldmann’ (unpublished PhD thesis: National Veterinary School of Lyon, 1951).

72

M. Fédida , G. Dannacher , M. Coudert and P. Belli , ‘Prophylaxie de la fièvre aphteuse, un succès ?’, Sciences vétérinaires-Médecine comparée, 94, 3 (1992), 207–225 .

73
*Ibid.*

74 CAC/FSV/19880158/101: Dossiers par départements: campagnes de vaccination anti-aphteuses, 1950-1956.

75
*Ibid.*

76 CAC/FSV/19880158/82: Réunion du Comité consultatif des épizooties, 5 novembre 1952; Intervention de Jean-Pierre Thiéry, *L’épizootie actuelle de fièvre aphteuse.*

77 Archives of the Pasteur Institute (AIP)/Fonds Gaston Ramon (GR)/boîte 20, pochette fièvre aphteuse: lettre des secrétaires perpétuels de l’Académie des sciences à Monsieur le ministre de l’Agriculture, 6 août 1952.

78 AIP/GR/boîte 20, pochette fièvre aphteuse: lettre de M. le Secrétaire perpétuel de l’Académie des sciences à Gaston Ramon, 9 août 1952.

79 JO, *Assemblée nationale*, Rapport de la commission de l’Agriculture, 3 juillet 1952, 1631.

80 CAC/FSV/19880158/105: Lettre de Camille Laurens à Édouard Herriot, 26 septembre 1952; et CAC/FSV/19880158/105-106: Remèdes proposés contre la fièvre aphteuse, 1949-1957.

81 CAC/FSV/19880158/100: Lettre du directeur des services sanitaires de la Mayenne à la FDSEA, juillet 1952.

82

G. Barrière , ‘Au sujet de la prophylaxie contre la tuberculose bovine’, Bulletin de l’Académie Vétérinaire de France, 31 (3 juillet 1958), 425–440, 430.

83 JO, *Assemblée nationale*, Séance du 11 juillet 1952, 3881.

84 Clément Bressou, « L’épizootie actuelle de fièvre aphteuse », *Comptes rendus des séances de l’Académie d’Agriculture de France*, séance du 19 novembre 1952, 38 (1952), 669–674, 672.

85 Their *in vitro* culture protocol had been developed by the Dutch Hermann Frenkel since 1947, enabling a large scale culture in 1951. See: Hermann S. Frenkel , ‘Research on Foot-and-Mouth Disease. III. The Cultivation of the Virus on a Practical Scale in Explantations of Bovine Tongue Epithelium’, American Journal of Veterinary Research, 12, 44 (1951), 187–190 14847110 .

86 Former CNEVA (centre national d’études vétérinaires et alimentaires – National Centre for Food and Veterinary Studies) Laboratory of Bovine Pathology.

87 Fedida *et al., op. cit.* (note 74).

88 Gaudillière, *op. cit.* (note 23), 185–186.

89

Félix Lucam et al., ‘Bilan de deux années de contrôle des vaccins anti-aphteux en France’, Bulletin de l’Office international des épizooties, 47, 11–12 (1957), 777–790 .

90 The same kind of invisibilisation of clinical results is described by Jean-Baptiste Fressoz about the Committee of the vaccine (Fressoz, *L’apocalypse joyeuse, op. cit.* [note 1]).

91 Lucam, *op. cit.* (note 91), 783–784.

92
*Ibid.*, 785.

93 This corresponds to the statistical table from Henderson’s test.

94 Lucam, *op. cit.* (note 91), 787.

95 Jas, *op. cit.* (note 58).

96 These breeders were exporting feeder cattle to the United Kingdom and wanted to be declared ‘free from FMD’ in the British sense.

97 CAC/FSV/19880158 /9: Circulaire interministérielle du 7 décembre 1955, prescrivant l’arrêt de toutes les opérations de dépistage dans la lutte contre la tuberculose bovine.

98 CAC/FSV/19880158 /93: Lettre de Marcel Quentin aux Présidents des sections syndicales départementales, 25th April 1957.

99 CAC/FSV/19880158 /66: Lettre de Marcel Quentin aux Présidents des sections syndicales départementales, 31st July 1957.

100

Madeleine Akrich , ‘Les formes de la médiation technique’, Réseaux, 60 (1993), 87–98 .

101 Decree n^o^ 61-694, 15^th^ May 1961, and Order of the 23 August 1961, with the exception of Finistère where stamping out remained the norm.

102 Joan H. Fujimura, “Constructing ‘Do-Able’ Problems in Cancer Research: Articulating Alignment”, *Social Studies of Science*, 17, 2, (1987), 257–293, 258.

103 Jas, *op. cit.* (note 58).

104

Allison Marie Loconto and David Demortain , ‘Standardization as Spaces of Diversity’, Engaging Science, Technology and Society, 3 (2017), 382–392, 383–384.

